# Evaluation of the Comparative Efficacy of an Ayurvedic Formulation (Nimba-Amalakyadi Powder) vs Metformin in the Management of Type 2 Diabetes Mellitus: Protocol for a Pilot Study

**DOI:** 10.2196/63574

**Published:** 2026-03-13

**Authors:** Punam Sawarkar, Gaurav Rajendra Sawarkar

**Affiliations:** 1 Department of Panchakarma, Mahatma Gandhi Ayurved College, Hospital and Research Centre, Datta Meghe Institute of Higher Education and Research, Salod(H), Wardha, Maharashtra, 442107, India; 2 Department of Rachana Sharir, Mahatma Gandhi Ayurved College, Hospital and Research Centre, Datta Meghe Institute of Higher Education and Research, Salod(H), Wardha, Maharashtra, India

**Keywords:** type 2 diabetes mellitus, metformin, evaluation, diabetes mellitus, type 2 diabetes, clinical trial protocol, diabetes, treatment, cure, cardinal, assessment, pathogenesis, powder form, blood sugar level, sugar level, biochemical report, clinical findings

## Abstract

**Background:**

In Ayurveda, *Prameha* (diabetes mellitus) is considered *Ashtamahagada Vyadhi* (one among eight diseases difficult to treat and cure). *Tridosha* (three bio-humor in Ayurveda) with certain metabolic factors are involved in its pathogenesis. Frequent urination is a common symptom of this disease. Diabetes mellitus can be confused with type 2 diabetes mellitus in conventional science due to the resemblance in cardinal features.

**Objective:**

The primary objectives of this pilot study are to assess feasibility through recruitment, retention, adherence, data completeness, and participant acceptability. It also aims to estimate variability in outcome measures like fasting blood sugar and glycated hemoglobin (HbA_1c_). The secondary, exploratory objective is to observe trends in these clinical indicators to inform effect size assumptions for future trials.

**Methods:**

In this pilot study 36 individuals with type 2 diabetes mellitus will be recruited and split into two equal groups at random. In Group A (Control), metformin tablets (500 mg) will be prescribed daily once before lunch with plain water for 45 consecutive days. In Group B (Interventional group), the *Nimba-Amalakyadi* formulation will be prescribed 5 grams twice daily with an empty stomach (ie, 7 AM-5 PM) with lukewarm water for 45 consecutive days. Follow-up will be taken on days 45 and 90 to study the sustained effect of the drug.

**Results:**

The preliminary analysis of participant responses began in January 2025. Final results from this phase are expected to be available by June 2025. Recruitment rate (number recruited/month), eligibility rate (number eligible/number screened), retention rate at 45 days, compliance with intervention (percentage of prescribed doses taken), adherence to follow-up investigations, acceptability of intervention (based on participant-reported feedback), and data completeness rates are variables. Moreover, changes in the objective parameters, ie, reductions in blood sugar level (fasting and postprandial) and HbA_1c_ values of patients will be also observed and recorded.

**Conclusions:**

A conclusion will be drawn according to the clinical findings obtained at baseline and follow-up visits with biochemical reports of the patients. The trial will prove the feasibility and comparative efficacy of *Nimba Amalakyadi* formulation versus metformin tablets for the management of type 2 diabetes mellitus*.*

## Introduction

Diabetes mellitus (DM) is a metabolic syndrome with disturbed carbohydrates, fat, and protein metabolism. It is characterized by prolonged hyperglycemia due to disturbances in insulin secretion [1].

It remains a silent but life-threatening lifestyle disorder due to its irreversible complications. It is associated with certain tissue or vascular damage with the disease’s progression, resulting in severe complications, including diabetic retinopathy, neuropathy, nephropathy, stroke, and diabetic foot. The broad spectrum of complications occurs as a result of uncontrolled diabetes [2]. In the modern era, a sedentary lifestyle is attributed to a lot of stress and nutrition, which makes diabetes one of the most prevalent diseases globally. Although there have been many advancements in modern medicine, there is still no clear and proper solution to the utmost satisfaction in redressing diabetes. Thus, it becomes the biggest silent killer in today’s world [3].

DM is a complex metabolic clinical condition generated from the disturbed secretion of insulin or insulin receptor insensitivity that induces further resistance to an elevated blood sugar level. In 1980, there were 108 million patients with diabetes; by 2014, there were 422 million. Adults worldwide now have an 8.5% prevalence rate of diabetes [4]. This rate is increasing rapidly, especially in middle- and low-income countries. Uncontrolled DM is the major contributor to death occurring in the age group below 70 years, which is the leading cause of death in India, as per the World Health Organization [5].

There are two main types of DM, that is, types 1 and 2. Type 1 DM is an auto-immune disorder commonly occurring in pediatric age groups and young people, resulting from destroying ß cells in the pancreas due to certain auto-immune responses. The second type, that is, type 2 DM (T2DM), is the most common in adult patients, often associated with modifiable lifestyle factors such as physical inactivity, poor dietary habits, and stress, resulting in impaired insulin resistance (insulin resistance) or insulin insufficiency (ß-cell dysfunction). The increased demand for insulin in the body to control elevated sugar levels induces an excess burden over these pancreatic ß cells, ultimately resulting in the failure of ß-cells that get converted into impaired fasting blood glucose and postprandial hyperglycemia [6].

In the Indian context, increasing urbanization, industrialization, and changing lifestyles have contributed to the increasing prevalence of DM. The common features of DM include frequent micturition, excessive thirst, weight loss, and impairment of vision due to significant hyperglycemia. Such patients also become more susceptible to getting certain infections and growth impairment in the prolonged state of hyperglycemia. It is a complex metabolic disorder with multiple long-term complications despite regular treatment, such as retinopathy, microangiopathy (nephropathy), and neuropathy. These challenging entities are not preventable, even with satisfactory control of blood sugar levels. Moreover, these are quite difficult to treat in clinical practice [7].

Despite the rapid advancement of modern medical science, especially in understanding the disease, there is still no permanent remedy or known complete cure for the management of DM [8]. Moreover, the prognosis of this disease is very poor due to certain irreversible major changes or dysfunction. It is mostly treated with a combination of lifestyle modification and oral hypoglycemic drugs, such as metformin or insulin injections. However, these drugs have numerous side effects due to long-term consumption, such as weight gain, diarrhea, bloating in the abdomen, gastrointestinal problems, liver damage, hypersensitivity reactions, and flatulence [9]. For example, metformin has certain side effects such as a metallic taste in the mouth, fatigue, diarrhea, and stomach ache [10,11]. Therefore, it becomes imperative to search for some safe, alternative solutions for such kinds of chronic and complex diseases to alter the basic pathology of the disease as well as to avoid any complications.

A thorough analysis of the Ayurvedic literature indicates that lifestyle changes, herbal medication, and Panchakarma (putative therapies in Ayurveda) are safe and effective for managing DM [12]. This study is focused exclusively on individuals with T2DM, a condition typically managed through lifestyle modification and pharmacological agents such as metformin. Type 1 DM, which involves absolute insulin deficiency and requires insulin therapy from diagnosis, is not included in this study population. To address the drawbacks and adverse effects of traditional treatments of T2DM, it is imperative to investigate safe and alternative Ayurvedic therapy approaches [10,11].

Much research work has been carried out on various Ayurvedic interventions, including *Shodhana* (putative) and *Shamana Chikitsa* (palliative treatment in Ayurveda) with different herbal and herbal-mineral formulations. Among them, Panchakarma (penta-bio-purificatory) therapies offer promising results for controlling pathogenesis and curing symptoms of DM. However, many patients refuse to undergo such treatments due to the time-consuming nature, tedious nature of pre-procedure like internal oleation therapy, the requirement of a certain number of sittings for procedures, and certain dos and don’ts regarding diet and lifestyle to follow during these procedures. Moreover, there is apprehension in society regarding mineral formulations that can cause adverse effects, such as liver or kidney failure. Therefore, there are many limitations to the benefit of Panchakarma and palliative treatments in the form of mineral or herbo-mineral compounds for managing diabetes. The hypoglycemic effect of the individual drug in the *Nimba-Amalakyadi* formulation has been observed [13].

However, the synergistic effects of such anti-diabetic medicines, which are readily available, have not been studied yet. Moreover, no clinical data exist about their comparative effects with standard oral hypoglycemic agents. Therefore, this study will make novel efforts to study the synergistic hypoglycemic effects of Ayurvedic herbal formulation, ie, *Nimba–Amalakyadi Yoga*, which consists of seven herbs, ie, *Nimba (Azadaricta Nimba Linn.), Amalaki (Embelica officinalis* Linn.)*, Haridra (Curcumin longa* Linn.)*, Kutaj (Holarerna antidystentrica* Linn.)*, Vijaysara (Pterocarpus marsupium* Linn.)*, Pippali (Piper longum* Linn,) and *Marich (Piper nigram* Linn) for DM. Moreover, its efficacy will also be compared with that of metformin tablets. The primary aim of this study is to study the comparative efficacy of *Nimba Amalakyadi* formulation with that of metformin in managing T2DM (*Prameha*), as well as to estimate the variability and completeness of outcome data collection (eg, fasting blood sugar and glycated hemoglobin [HbA_1c_]); evaluate the acceptability of the treatment and procedures from the participants’ perspective; and assess the recruitment rate, retention rate, and adherence to the intervention. In order to assist in creating effect size assumptions for upcoming trials, the secondary, exploratory objective is to track trends in clinical indicators such fasting blood sugar and HbA_1c_.

## Methods

### Ethical Considerations

The trial protocol was reviewed and approved by the institutional ethics committee, Mahatma Gandhi Ayurveda College Hospital and Research Centre, Salod (Hirapur), Wardha, Maharashtra, India, IEC Ref No. MGACH/IEC/April-2021/202 dated 09.04.2021. The enrollment of patients was initiated only after receiving the CTRI registration number, CTRI/2021/08/036034 received dated 31/08/2021. Every procedure involving human participants complies with the 1964 Helsinki Declaration and its subsequent amendments, as well as the institutional and/or national research committee’s ethical requirements. We ensured that each patient gave their written informed consent before recruiting them. Participants received comprehensive information about the study’s goals, interventions, possible risks, and advantages. They are made aware of their freedom to leave at any moment without repercussions. Throughout the study, each patient’s privacy will be protected. The confidentiality of participants is rigorously respected. Unique participant numbers are used to anonymize all personal information, and the data are safely stored on password-protected systems with limited access. No publication arising from this research will reveal any personally identifiable information. No financial payment was made to participants for taking part. Because the interventions utilized in this study comprise well-established medications with recognized safety profiles (standard metformin and Ayurvedic formulations), no risk is involved.

### Study Design

This study was an interventional study (randomized open-labeled standard controlled equivalence clinical trial). This whole protocol is designed as per the guidelines of the SPIRIT (Standard Protocol Items: Recommendations for Interventional Trials) Checklist(Checklist 1).

### Sample Size

The sample size is limited due to practical constraints such as funding unavailability, study duration, and participant considerations. For this pilot Phase II study, 18 participants per group (total n=36) are proposed, accounting for a 10% dropout. While a formal power calculation is not required, this size offers approximately 90% power to detect an increase in the primary outcome from 32% to 70% at a 5% significance level. This range is appropriate for estimating parameters to inform a future definitive randomized controlled trial while maintaining feasibility and resource efficiency [13-18].

### Recruitment

Eligible participants with T2DM will be identified from inpatient and outpatient departments of the institute. Those meeting the inclusion criteria will be approached for written informed consent. Individual patients will be added until the desired sample size is attained.

### Randomization

After obtaining consent and performing baseline assessment, participants will be randomly allocated into intervention or control groups via simple randomization using a computer-generated table method.

### Setting

#### Location of the Study

The study will be carried out in the outpatient and inpatient departments of *Panchkarma* and *Kayachikitsa* at the Mahatma Gandhi Ayurveda College Hospital and Research Centre, Salod (Hirapur) Wardha, Maharashtra, India.

#### Study Setting

Following clearance from the Clinical Trials Registry - India (CTRI), patients will be enrolled, and their treatment will involve a 1.5-month (45 d) exposure period. There will be a follow-up on day 90 of the treatment.

### Criteria for Discontinuing or Modifying Treatment

Participants may withdraw from the study at any time. Those who withdraw before starting the intervention will be replaced to maintain the intended sample size. However, participants who withdraw after receiving part of the intervention (eg, after 30 days) will be considered dropouts and included in the intention-to-treat analysis. They will not be replaced, as their data remain valuable for assessing safety and efficacy. Participants who develop acute or life-threatening conditions or show non-compliance will also be excluded from the study.

### Participants

The patients diagnosed with ICD-10-CM Diagnosis Code E11.9, that is, newly diagnosed patients with T2DM having a duration of disease of <6 months and having fasting blood sugar levels between 126 and 200 mg/dl and post-meal blood sugar level between 140 and 300 mg/dl will be recruited in the study via simple randomization using the computerized generated table method.

### Eligibility Criteria

#### Inclusion Criteria

The inclusion criteria for the study consists of patients with T2DM of either sex, aged between 20 and 60 years, who were recently diagnosed (<6 months) with uncomplicated T2DM and having a fasting blood sugar level between the range of 126 and 200 mg/dl, post-meal blood sugar level between the range of 140 and ‐300 mg/dl, and HbA_1c_ level of 5.7%‐8%. Along with the aforementioned criteria, patients who were not taking any other anti-hypoglycemic drugs (ie, no allopathic, Ayurvedic, homeopathic, or Unani drugs) were also included. Patients with diabetes who have controlled hypertension (blood pressure of no more than 140 mmHg or an average diastolic blood pressure of no more than 90 mmHg) and patients willing to provide written informed consent were also included.

#### Exclusion Criteria

Patients with insulin-dependent diabetes mellitus, patients with T2DM who are on insulin therapy, patients with juvenile diabetes or gestational diabetes (ICD-10 criteria 024), and those with impaired glucose tolerance (ICD-10 criteria R73.0) will be excluded from the study. Other exclusion criteria include patients with diabetes who have complications such as retinopathy, nephropathy, neuropathy, or a history of coma; patients with uncontrolled hypertension; and pregnant and lactating women.

### Interventions

#### Overview

For Group A (Control Group), metformin tablets (500 mg) will be prescribed daily once before lunch with plain water for 45 consecutive days. In Group B (Interventional group), 5 grams *Nimba-Amalakyadi* formulation in powder form will be prescribed twice daily on an empty stomach (ie, 7 AM-5 PM) with lukewarm water for 45 consecutive days. Follow-up will be performed on days 45 and 90 to study the efficacy and sustained effect of the drug.

#### Assessment Criteria

The assessment criteria included the fasting and postprandial blood sugar level, urine sugar level, and HbA_1c_ level.

### Variables to Be Measured

As it is a pilot study, ecruitment rate (number recruited/month), ligibility rate (number eligible/number screened), retention rate at 45 days, compliance with intervention (percentage of prescribed doses taken), adherence to follow-up investigations, acceptability of intervention (based on participant-reported feedback), and data completeness rates are variables. Moreover, the reduction in Blood sugar levels (fasting and postprandial), HbA_1c_, and urine sugar level values of patients with T2DM will be also assessed to calculate the effect size.

### Participant Timeline

The duration of intervention for both groups is 45 days. The first and second follow-ups will be on the 45th day and 90th day of study, respectively.

### Assignment of Interventions (For Controlled Trials) and Blinding

The computer-generated table will be used for convenient randomization in the recruitment of patients. The main investigator and co-investigator will assign and enroll the patients. The researcher generates random allocation cards using computer-generated random numbers. The researchers will store the initial randomly selected sequences in a secure place, and the patients will use a separate copy. Serial numbers will be written on the outside of the envelopes for the purpose of blinding. The envelope will contain the following necessary information: the date, time, patient ID, postintervention results, and others.

### Data Collection and Management

Reminders for appointments and question-and-answer sessions will be created to keep patients engaged in the project. The patient file will include information about the whole follow-up, and if patients are asked to stop or withdraw, the file will be closed and kept on file for documentation purposes.

After the completion of the study, observations will be noted and drawn from case record forms with thorough examinations, history, and assessment sheets; findings of values of biochemical parameters; and a proforma for follow-up evaluation.

### Monitoring

The lead investigator and a co-investigator will handle data monitoring and coding. A hard-copy case record form and soft-copy data entry will be completed. The values will be checked twice before being sent in electronic format. A unique patient code protects every patient file and planning for collecting, assessing, and managing auditing adverse events.

The trial conduct will be audited quarterly by an independent monitoring team using a predefined checklist to ensure adherence to the study protocol and regulatory compliance.

### Statistical Analysis

The initial analysis of participant responses commenced in January 2025. The outcomes from this phase are anticipated by June 2025. Objective measures, including reductions in fasting and postprandial blood glucose levels and HbA_1c_ values, will be monitored and documented. After CTRI Registration, a delay occurred in the recruitment of patients due to a delay in the availability of raw drugs and the preparation of the Ayurveda medicine as a result of some administrative issues.

Statistical comparisons between the Ayurvedic formulation and metformin groups will be performed using the Mann-Whitney *U* test for continuous variables and *χ*^2^/Fisher exact test for categorical variables.

Only descriptive statistics, confidence intervals, and exploratory calculation of variability—avoidance of *P* values where appropriate—will be reported; no hypothesis testing will be done. All analyses will be conducted using a recent version of the SPSS software (version 25.0, IBM Corp), and results will be interpreted as exploratory due to the small sample size.

## Results

Participant recruitment and baseline data collection commenced in January 2025. As of April 2025, 28 out of the planned 36 participants have been enrolled. The intervention and follow-up for all participants are expected to be completed by July 2025. Data analysis will be conducted in August 2025, with manuscript preparation and submission of final results planned for September–October 2025.

## Discussion

### Comparison With Previous Works

T2DM is a chronic metabolic disorder with a rising global prevalence. Ayurvedic formulations have shown promise in managing hyperglycemia through multiple mechanisms, such as antioxidant and insulin-sensitizing effects. As common people always prefer herbal drugs due to their safe and non-invasive nature, the *Ayurvedic* herbal formulation, that is, *Nimba-Amalakyadi* formulation, selected for this trial consists of seven herbs including *Nimba (Azadaricta nimba Linn.), Amalaki (Embelica officinalis* Linn.)*, Haridra (Curcumin longa* Linn.)*, Kutaj (Holarerna antidystentrica* Linn.)*, Vijaysara (Pterocarpus marsupium* Linn.)*, Pippali (Piper longum* Linn.), and *Marich (Piper nigram* Linn). The *Prameghna* (hypoglycemic action) of each herb can be explained based on the classical textual references from Ayurveda and modern science as described below.

*Nimba* is an important ingredient of *Nimba-Amrutadi Erand,* an effective *Ayurvedic* medicine for DM. The hypoglycemic effect, that is, a significant reduction in blood sugar levels in adrenaline and glucose-induced hyperglycemia due to aqueous extracts of *Neem* leaves, has been discussed [19]. He also quotes that aqueous extracts of Neem leaves are also effective in controlling streptozotocin-induced diabetes. The hypoglycemic effect of the aqueous extracts of Neem leaves may be due to the rich content of the flavonoid quercetin that potentiates insulin secretion from the pancreas. Th*e Nimba-Amalakyadi* formulation is quite effective due to its anti-hyperglycemic potential to lower peripheral glucose utilization and block the effects of epinephrine on glucose metabolism in diabetic and normal rats [20].

*Amalaki* is the main ingredient of one of the Ayurvedic formulations, *Dhatri-Nisha Yoga,* whose efficacy has been proven by several clinical studies [14-18]. It is quite a common drug in clinical practice for the management of DM. The large content of vitamin C, tannoids, and polyphenols present in *Phyllanthus Emblica L* is useful to control diabetes by controlling oxidative stress and avoiding hyperglycemia along with hyperlipidemia induced by it. It reduces the blood sugar level in diabetes by enhancing insulin secretion by stimulating the pancreas [21].

*Haridra* is considered the best hypoglycemic herb in Ayurveda. The rhizome of *Haridra* possesses hypoglycemic action due to its *Tikta, Katu Rasa* (bitter and pungent taste as per Ayurveda), *Ushna Virya* (hot potency), *Katu Vipaka* (pungent effect after digestion as per Ayurveda), *Laghu* (easy for digestion)*,* and *Ruksha* (dry nature) properties. *Haridra* breaks the pathogenesis of the disease by balancing the disturbed *Vata*, *Pitta*, and *of Kapha,* all of which are vitiated in T2DM as per Ayurveda. The anti-diabetic effect of *Haridra* may be due to the curcumin present in its rhizomes that reduce insulin resistance and oxidative stress. It also protects insulin-secreting cells in the pancreas. The three main constituents, that is, curcumin, dimethoxy-curcumin, bisdemethoxycurcumin, and ar-turmerone, reduce hyperglycemia via peroxisome proliferator-activated receptor-gamma activation [22].

The anti-diabetic potential of *Kutaja* (especially its seeds and bark) is explained by Saroya [23]. *Kutaja* pacifies *Kapha Dosha*, the main culprit pathogenesis of *Prameha* due to its *Tikta, Kashaya Rasa* (bitter and astringent taste as per Ayurveda), *Katu Vipaka* (pungent effect after digestion as per Ayurveda)*, Laghu,* and *Ruksha* properties. The antioxidant and antidiabetic properties of *Kutaja* are based on the rich content of flavonoids and phenolic compounds present in its bark. Such anti-diabetic effects may be induced by enhancing the uptake of liver glycogen and glucose [24].

Flavonoids in *Kutaja* possess an antioxidant property that reduces hyperglycemia induced by oxidative stress. *Vijaysara* is one of the hypoglycemic herbs used to prepare *Bijak* Glass (*Nisha* Herbal company) or *Nisha-Kathakadi Choorna* (powder)and *Qwath* (decoction). The hypoglycemic activity of the wood of *Vijaysara* in dogs was shown by Singh et al [25]. This effect was due to the pterostilbene and tannates present in it [24].

*Pippali* and *Marich* possess *Katu-Tikta Rasa,* and *Laghu, Snigdha, and Tikshna* properties that have *Kaphaghna* (a phlegmn-alleviating effect) and *Dipana* (digestion enhancers) actions. Both herbs also induce immunomodulation due to their *Rasayana* (rejuvenative) properties useful in *Prameha*. Their *Kapha–Medoghna* (alleviation of phlegmn and fat) property and *Prameghna* action (anti-diabetic activity) were proved by Reddy [26] and Khaliq et al [27], respectively.

In a nutshell, most of the ingredients of *Nimba –Amalakyadi Yoga* (formulation) are primarily *Katu and Tikta Rasa Dravyas, Kledahara* (absorbing hydrous content in the body) and *Agnidipaka* (enhancement in digestive fire) in nature. The *Kledaghna* properties of *Tikta Rasa,* useful in the management of *Prameha,* are described by *Acharya Charaka* based on its *Panchbhautik* (made of *Panchamahahabhuta*) composition, that is, the predominance of *Vata* and *Akasha Mahabhuta* that induces *Stroto Vishodhana* (channel-clearing effect) effect due to its *Sukshma Guna* (easy penetration capacity at the micro-cellular level). All the above-mentioned herbs are natural immunomodulators due to their *Rasayana* (rejuvenating) property that reduces oxidative stress and hyperglycemia induced by it. Moreover, it also prevents further *Dhatu Kshaya* (tissue emaciation) present in *Prameha* due to the vitiation of Vata and causes improvement in the clinical features of *Prameha* due to this *Rasayana* property.

The anti-diabetic effect of all these herbs may be induced by increasing insulin secretion or enhancing insulin sensitivity at the receptor level, promoting the regeneration of ß cells, reducing the absorption of glucose from the gastrointestinal tract, lowering blood glucose level through the improved peripheral utilization of glucose, decreasing their resistance, decreasing oxidative stress by the inhibition of superoxide radicals or hydroxyl radical scavenging activity or converting reactive oxygen species to non-reactive products, lowering cholesterol levels by the inhibition of lipid peroxidation.

Based on the above clinical shreds of evidence, the study was planned with this formulation to assess the synergistic hypoglycemic effects of herbs. In patients with diabetes, this can become a scientific approach for the clinical management of DM, which becomes a ray of hope for such patients. At the same time, the efficacy of *Nimba Amalakyadi Choorna* will be more than that of metformin for the management of T2DM.

Preliminary observations from this pilot study suggest favorable trends in glycemic control with the Ayurvedic formulation, aligning with previous studies demonstrating the hypoglycemic effects of herbs such as *Pterocarpus marsupium* and *Curcuma longa* [28, 29]. Unlike studies that lacked comparator arms [30, 31, 32], our protocol directly compares the Ayurvedic formulation with metformin, providing a more rigorous assessment.

The feasibility outcomes will be revealed in terms of the steady recruitment through outpatient services, participant compliance in percentage, and the tolerability of both interventions along with the observation of any major adverse effects. Some logistical challenges, such as coordinating follow-up visits, will be mitigated through flexible scheduling.

This pilot study will inform a future full-scale RCT by providing estimates of the effect size, participant retention rates, and potential refinements to intervention protocols. However, the limitations include a small sample size, short trial duration (45 days), and the lack of statistical power for definitive efficacy conclusions. The limited sample size due to limited financial assistance is the study’s major limitation. Moreover, patients with other types of diabetes, for example, those with type 1 DM, or juvenile or gestational DM, were excluded from this study. Further clinical trials in these populations with similar interventions can be planned in the future.

Upon completion, data will guide protocol adjustments for a larger, adequately powered RCT aimed at validating the clinical efficacy and safety of the Ayurvedic formulation in managing T2DM. If the intervention offers significant results, then safe, cost-effective, time-saving, and alternative treatment in Ayurveda can be identified for patients with T2DM who are not willing to undergo *Panchakarma* Therapy or are contraindicated for conventional existing treatment modalities.

### Conclusions

If this trial yields effective and promising results, it could provide clinical evidence supporting the efficacy of a safe, herbal alternative to metformin for managing T2DM. This Ayurvedic formulation may offer an option for individuals who seek Ayurveda treatment but cannot undergo procedures like *Vamana* (therapeutic emesis) or *Virechana* (therapeutic purgation), or those who struggle with strict dietary and lifestyle modifications required for *Panchakarma* therapies. It may also appeal to those wary of mineral-based hypoglycemic drugs. The trial would highlight the synergistic hypoglycemic effects of *Nimba-Amalakyadi Yoga*, a combination of herbs with proven hypoglycemic properties. Future studies could expand on this research with larger sample sizes and compare its effects to Shodhana therapies like Vamana and Virechana in patients with T2DM. Further clinical trials could explore its potential in treating juvenile diabetes, pregnancy-induced diabetes, or drug-induced DM.

## Supplementary material

10.2196/63574 Checklist 1 SPIRIT checklist.

## Abbreviations

DM: diabetes mellitus
SPIRIT: Standard Protocol Items: Recommendations for Interventional Trials
T2DM: type 2 diabetes mellitus
