# Correction: Evaluation of the Comparative Efficacy of an Ayurvedic Formulation (Nimba-Amalakyadi Powder) vs Metformin in the Management of Type 2 Diabetes Mellitus: Protocol for a Pilot Study

**DOI:** 10.2196/96344

**Published:** 2026-04-09

**Authors:** Punam Sawarkar, Gaurav Rajendra Sawarkar

**Affiliations:** 1 Department of Panchakarma, Mahatma Gandhi Ayurved College, Hospital and Research Centre, Datta Meghe Institute of Higher Education and Research, Salod(H), Wardha, Maharashtra, 442107, India; 2 Department of Rachana Sharir, Mahatma Gandhi Ayurved College, Hospital and Research Centre, Datta Meghe Institute of Higher Education and Research, Salod(H), Wardha, Maharashtra, India

In “Evaluation of the Comparative Efficacy of an Ayurvedic Formulation (*Nimba-Amalakyadi* Powder) vs Metformin in the Management of Type 2 Diabetes Mellitus: Protocol for a Pilot Study” [1], the authors made one addition.

The following institution name was added to both affiliations:

Datta Meghe Institute of Higher Education and Research

The correction will appear in the online version of the paper on the JMIR Publications website, together with the publication of this correction notice. Because this was made after submission to PubMed, PubMed Central, and other full-text repositories, the corrected article has also been resubmitted to those repositories.
