# Supplementary material for: Evaluation of the Comparative Efficacy of an Ayurvedic Formulation (Nimba-Amalakyadi Powder) vs Metformin in the Management of Type 2 Diabetes Mellitus: Protocol for a Pilot Study
Source: JMIR Res Protoc. 2026 Mar 13;15:e63574. doi: 10.2196/63574 (PMC12987602; doi:10.2196/63574)
Supplement: Checklist 1 [file resprot-v15-e63574-s001.pdf]

SPIRIT 2013 Checklist: Recommended items to address in a clinical trial protocol

Section/Item	Item No.	Description	Page No.
**Administrative Information**			
Title	1	Descriptive title including trial acronym	1
Trial registration	2a	Trial identifier and registry name	2
Trial registration: data set	2b	All items from WHO Trial Registration Data Set	2
Protocol version	3	Date and version identifier	13
Funding	4	Sources and types of financial, material, and other support	13
Roles and responsibilities	5a	Names, affiliations, and roles of protocol contributors	13
	5b	Name and contact info for trial sponsor	13
	5c	Role of sponsor/funder in study design, etc.	13
**Introduction**			
Background and rationale	6a	Description of research question and justification	3
	6b	Explanation for choice of comparators	4
Objectives	7	Specific objectives or hypotheses	5
Trial design	8	Description of trial design (e.g., parallel, factorial)	6
**Methods: Participants, Interventions, Outcomes**			
Study setting	9	Description of study settings and sites	6
Eligibility criteria	10	Inclusion and exclusion criteria for participants	7
Interventions	11a	Interventions for each group, including dose, mode, etc.	7
	11b	Criteria for discontinuing/modifying allocated interventions	6
	11c	Strategies to improve adherence	6
	11d	Relevant concomitant care permitted/prohibited during trial	9
Outcomes	12	Primary, secondary, and other outcomes specified	9
Participant timeline	13	Time schedule of enrolment, interventions, assessments	9
Sample size	14	Estimated number of participants and rationale	6

Recruitment	15	Strategies for achieving adequate enrolment	6
**Methods: Assignment of interventions (for controlled trials)**			
Allocation: sequence generation	16a	Method of random sequence generation	6
Allocation concealment mechanism	16b	Mechanism of implementing the allocation	6
Implementation	16c	Who generates the sequence, enrolls and assigns	6
Blinding	17a	Who is blinded after assignment and how	6
	17b	Circumstances under which unblinding is permissible	-
**Methods: Data collection, management, and analysis**			
Data collection methods	18a	Plans for assessment and collection of data	9
	18b	Plans to promote participant retention	9
Data management	19	Plans for data entry, coding, and security	9
Statistical methods	20a	Statistical methods for analyzing outcomes	10
	20b	Methods for additional analyses (e.g., subgroup)	10
	20c	Definition of analysis population and handling of missing data	10
**Monitoring**			
Data monitoring	21a	Composition and role of the data monitoring committee	9
	21b	Description of interim analyses and stopping guidelines	9
Harms	22	Plans for collecting, assessing, and managing AEs	9
Auditing	23	Frequency and procedure for auditing trial conduct	10
**Ethics and Dissemination**			
Research ethics approval	24	Plans for seeking ethics approval	5
Protocol amendments	25	Plans for communicating important protocol changes	5
Consent or assent	26a	Who will obtain informed consent and how	5
	26b	Additional consent for use of data/specimens	5
Confidentiality	27	How personal information will be collected and stored	5

Declaration of interests	28	Financial and other competing interests	13
Access to data	29	Who will have access to the final dataset	13
Ancillary and post-trial care	30	Provisions for post-trial care and compensation	14
Dissemination policy	31a	Plans for publication and sharing of results	14
	31b	Authorship guidelines	14
	31c	Public access to full protocol, data, and results	14
